# Machine learning-based prediction of NAFLD in patients with type 2 diabetes using routine clinical and biochemical indicators

**DOI:** 10.3389/fendo.2026.1761846

**Published:** 2026-04-13

**Authors:** Yongjun Zeng, Jinlin Wang, Kun Lin, Lu Ao, Chenggong Zhu, Qiang Yi, Ya Fu, Yongbin Zeng

**Affiliations:** 1 Department of Cardiovascular, The First Affiliated Hospital, Fujian Medical University, Fuzhou, China; 2 Department of Cardiovascular, National Regional Medical Center, Binhai Campus of The First Affiliated Hospital, Fujian Medical University, Fuzhou, China; 3 Department of Laboratory Medicine, Gene Diagnosis Research Center, The First Affiliated Hospital, Fujian Medical University, Fuzhou, China; 4 Department of Laboratory Medicine, National Regional Medical Center, Binhai Campus of the First Affiliated Hospital, Fujian Medical University, Fuzhou, China; 5 Fujian Key Laboratory of Laboratory Medicine, The First Affiliated Hospital, Fujian Medical University, Fuzhou, China; 6 Department of Bioinformatics, Fujian Key Laboratory of Medical Bioinformatics, School of Medical Technology and Engineering, Fujian Medical University, Fuzhou, China

**Keywords:** clinical indicators, machine learning, non-alcoholic fatty liver disease, risk prediction, type 2 diabetes mellitus

## Abstract

**Objectives:**

This study aimed to develop and evaluate machine learning (ML) models for predicting non-alcoholic fatty liver disease (NAFLD) in patients with type 2 diabetes mellitus (T2DM) using readily accessible clinical and biochemical indicators.

**Methods:**

A total of 2,459 patients with T2DM were enrolled in this cross-sectional study. Eight ML algorithms, logistic regression (LG), k-nearest neighbors (k-NN), support vector machine (SVM), decision tree (DT), random forest (RF), gradient boosting machine (GBM), extreme gradient boosting (XGBoost), and naïve Bayes (NB), were developed to construct predictive models. Feature selection was performed using Boruta, recursive feature elimination, and LASSO regression. Model performance was assessed using several metrics, including the area under the receiver operating characteristic curve (AUC), accuracy, recall, F1 score, and decision curve analysis.

**Results:**

Among the study population, 1,309 individuals (53.23%) were diagnosed with NAFLD. Sixteen variables, including BMI, waist circumference, systolic blood pressure, triglycerides, HDL-C, ALT, GGT, bilirubin fractions, albumin, BUN, GFR, fasting insulin, RBC, and hemoglobin, were selected as key predictors. The SVM model demonstrated the best overall performance, achieving an AUC of 0.920, accuracy of 0.839, and specificity of 0.898 in the training set, and an AUC of 0.833 and accuracy of 0.733 in the validation set. Decision curve analysis confirmed superior clinical utility of the SVM model compared with other algorithms.

**Conclusions:**

ML-based models, particularly the SVM algorithm, effectively predicted NAFLD among patients with T2DM using easily accessible clinical and biochemical indicators. These findings highlight the potential utility of ML-assisted screening tools for improving early identification and risk stratification of NAFLD in diabetic populations.

## 1 Introduction

Non-alcoholic fatty liver disease (NAFLD) is characterized by excessive hepatic fat accumulation exceeding 5% of liver weight in the absence of significant alcohol intake (1). As the disease progresses, simple steatosis may advance to non-alcoholic steatohepatitis (NASH), liver fibrosis, cirrhosis, and even hepatocellular carcinoma (HCC). NAFLD is now recognized as one of the most common chronic liver diseases worldwide, affecting nearly 25% of the global adult population, with an even higher prevalence reported in Asian countries (2). NAFLD frequently coexists with components of metabolic syndrome, including obesity, hyperlipidemia, hypertension, and type 2 diabetes mellitus (T2DM), reflecting a shared metabolic pathophysiology.

T2DM is a complex metabolic disorder caused by impaired insulin secretion and/or insulin resistance and has become a major public health burden globally (3). Currently, approximately one in eleven adults lives with T2DM, a condition that affects not only pancreatic β-cell function but also multiple organs, including the liver, kidneys, heart, adipose tissue, and central nervous system (4, 5). The systemic metabolic dysregulation associated with T2DM leads to severe long-term complications and markedly increases morbidity and mortality. Importantly, NAFLD and T2DM exhibit a bidirectional interaction: T2DM accelerates the progression of NAFLD to advanced fibrosis and cirrhosis, while NAFLD further exacerbates insulin resistance and increases the risk of developing T2DM (6). This interplay underscores the clinical necessity for early identification of NAFLD in patients with T2DM.

Although liver biopsy remains the diagnostic gold standard for NAFLD, its invasiveness, potential complications, high cost, and sampling variability limit its suitability for routine or large-scale screening (7). Abdominal ultrasonography, the most commonly used non-invasive alternative, also faces substantial limitations, including reduced sensitivity for mild steatosis and strong operator dependence. As a result, accurately identifying NAFLD in high-metabolic-risk populations, particularly patients with T2DM, remains challenging in routine clinical practice. These limitations highlight the urgent need for simple, objective, and accurate prediction tools based on routinely available clinical and biochemical indicators that can be applied across various healthcare settings. Machine learning (ML) techniques, which model nonlinear patterns and high-dimensional interactions, provide powerful tools for enhancing NAFLD risk prediction. In this study, we developed and compared multiple ML models to predict NAFLD in patients with T2DM using easily accessible clinical and biochemical parameters.

## 2 Participants and methods

### 2.1 Ethics

This study was approved by the Ethics Committee of the First Affiliated Hospital of Fujian Medical University (approval number: MTCA, ECFAH of FMU [2021] 047). All procedures involving human participants were performed in accordance with the ethical principles of the Declaration of Helsinki and its later amendments. Written informed consent was obtained from all participants prior to enrollment.

### 2.2 Study design and population

This cross-sectional study included 2,459 patients with type 2 diabetes mellitus (T2DM) who were hospitalized in the Department of Endocrinology at the First Affiliated Hospital of Fujian Medical University between January 2018 and September 2023.

Inclusion Criteria: (1) A confirmed diagnosis of T2DM based on the 2022 American Diabetes Association (ADA) criteria.

Exclusion Criteria: (1) Type 1 diabetes or other specific forms of diabetes, including latent autoimmune diabetes in adults (LADA). (2) History of viral hepatitis (e.g., hepatitis B surface antigen positivity or hepatitis C infection), drug-induced liver injury, autoimmune liver diseases, hepatic cirrhosis, or hepatocellular carcinoma. (3) Newly diagnosed cirrhosis or hepatocellular carcinoma during hospitalization. (4) Active COVID-19 infection or other severe acute infections. (5) Incomplete clinical or laboratory data, including absence of key biochemical indicators or >10% missing data. (6) Systemic malignancies or ongoing radiotherapy/chemotherapy. (7) Absence of abdominal Doppler ultrasonography required for NAFLD assessment. (8) Excessive alcohol consumption (>140 g/week for men or >70 g/week for women), in order to exclude alcoholic fatty liver disease.

NAFLD definition: NAFLD was diagnosed according to the 2018 Guidelines for the Prevention and Treatment of Nonalcoholic Fatty Liver Disease issued by the Chinese Society of Hepatology (8), and was set as the primary outcome variable in this study.

**Figure 1:**
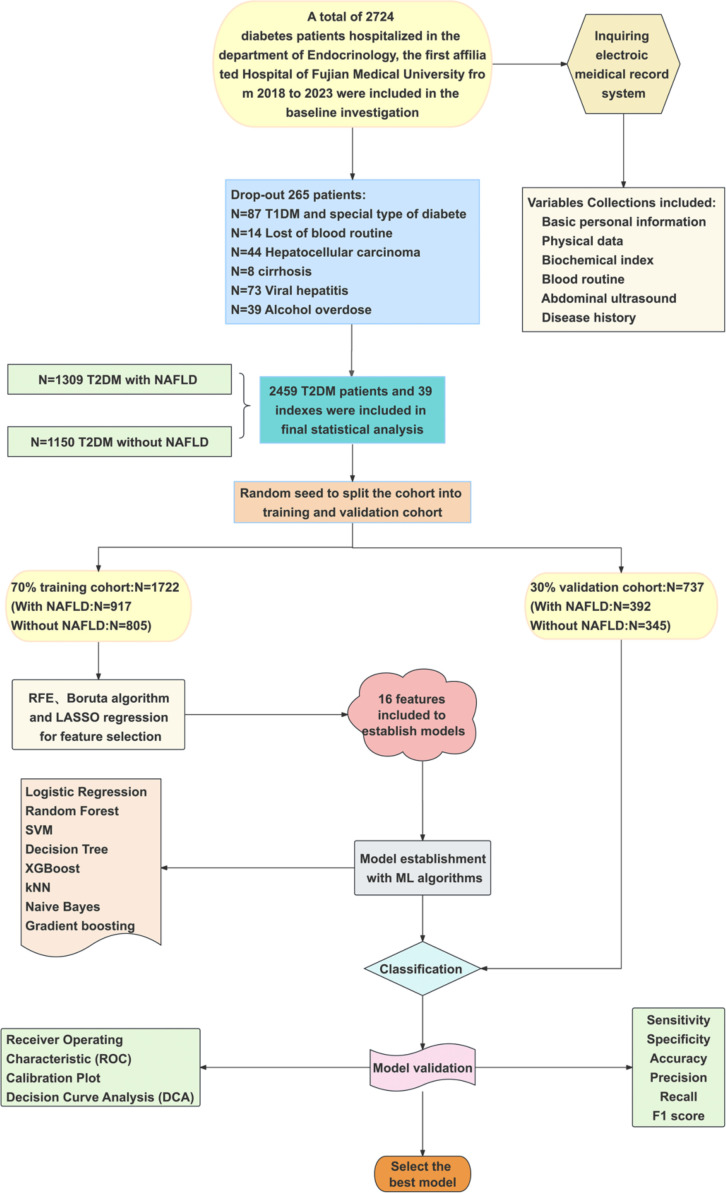
Flowchart of the study participant selection and analysis process.

A detailed flowchart of participant selection and analytical procedures is presented in **Figure 1**.

### 2.3 Clinical measurements

General demographic and anthropometric information collected included sex, age, height, weight, body mass index (BMI; calculated as weight in kilograms divided by height in meters squared), waist circumference (WC), systolic blood pressure (SBP), diastolic blood pressure (DBP), smoking status, and alcohol consumption.

Laboratory measurements encompassed a comprehensive metabolic, hepatic, renal, and hematologic panel, including total cholesterol (TC), triglycerides (TG), high-density lipoprotein cholesterol (HDL-C), low-density lipoprotein cholesterol (LDL-C), alanine aminotransferase (ALT), aspartate aminotransferase (AST), alkaline phosphatase (ALP), gamma-glutamyl transferase (GGT), total bilirubin (TBIL), direct bilirubin (DBIL), total protein (TP), albumin (ALB), globulin (GLB), uric acid (UA), blood urea nitrogen (BUN), serum creatinine (Scr), glomerular filtration rate (GFR), fasting blood glucose (FBG), glycated hemoglobin (HbA1c), fasting insulin (FI), white blood cell count (WBC), red blood cell count (RBC), monocyte percentage (M%), hemoglobin (Hb), and platelet count (PLT).

Past medical history was reviewed, including the presence of hypertension, cardiovascular or cerebrovascular diseases, respiratory diseases, and diabetes-related complications.

### 2.4 Feature selection and performance of machine learning algorithms

#### 2.4.1 Feature selection in the training set

A total of 39 candidate variables were initially included. Based on Spearman correlation analysis, four variables-height, weight, globulin, and serum creatinine-were removed because they exhibited correlation coefficients >0.6 with other variables, leaving 35 variables for downstream feature selection.

Three feature selection methods were applied in the training set: Recursive Feature Elimination (RFE), the Boruta algorithm, and the Least Absolute Shrinkage and Selection Operator (LASSO) regression, implemented using the caret, Boruta, and glmnet R packages, respectively. The intersection of the selected features from all three methods was considered the most robust set of predictors. The overlap was visualized using the VennDiagram R package.

#### 2.4.2 Model construction

Eight machine learning algorithms were developed and compared using the caret R package: Logistic Regression (LG), k-Nearest Neighbors (k-NN), Support Vector Machine (SVM), Decision Tree (DT), Random Forest (RF), Gradient Boosting Machine (GBM), Extreme Gradient Boosting (XGBoost), and Naïve Bayes (NB).

To reduce the risk of overfitting, model hyperparameters were optimized through randomized search combined with 10-fold cross-validation repeated five times. Optimized models were then trained on the training dataset and subsequently evaluated in the testing dataset. Model performance was assessed using the following evaluation metrics including sensitivity, specificity, accuracy, area under the receiver operating characteristic curve (AUC), positive predictive value (PPV), negative predictive value (NPV), positive likelihood ratio (LR+), and negative likelihood ratio (LR-), recall, and F1 score. Clinical utility was further examined using Decision Curve Analysis (DCA), which quantified the net benefit across a range of threshold probabilities.

### 2.5 Statistical analysis

All statistical analyses were performed using R software (version 4.3.1; http://www.R-project.org). The normality of continuous variables was evaluated using the *Shapiro-Wilk* test. Normally distributed variables are presented as mean ± standard deviation (SD), whereas non-normally distributed variables are summarized as median with interquartile range (IQR). Differences between two groups were assessed using the independent samples t-test for normally distributed variables or the *Wilcoxon rank-sum* test for non-normally distributed variables.

Categorical variables were expressed as counts and percentages, and group differences were examined using the *Chi-square* test or *Fisher’s exact* test, as appropriate. Comparisons of AUC values between machine learning models were conducted using the DeLong test.

Machine learning analyses were implemented using the caret, Boruta, glmnet, pROC, and VennDiagram R packages, among others. A two-sided p-value < 0.05 was considered statistically significant.

## 3 Results

### 3.1 Demographic and clinical characteristics of study participants

A total of 2,459 patients with T2DM met the inclusion and exclusion criteria (**Figure 1**). Among them, 1,309 (53.23%) were diagnosed with NAFLD, including 773 males (59.1%). Participants were randomly divided into a training set (n = 1,722) and a validation set (n = 737) at a ratio of 7:3.

**Table 1** depicts the clinical baseline characteristics of T2DM patients based on the presence or absence of NAFLD. Compared to patients without NAFLD, those with both NAFLD and T2DM exhibited higher values in BMI (25.34 kg/m² vs. 22.50 kg/m²), SBP (133.00 mmHg vs. 131.00 mmHg), TG (1.66 mmol/L vs. 1.16 mmol/L), FBG (7.30 mmol/L vs. 6.96 mmol/L), ALT (20.00 U/L vs. 16.00 U/L), AST (18.00 U/L vs. 17.00 U/L), GGT (26.00 U/L vs. 18.00 U/L), DBIL (3.60 μmol/L vs. 3.00 μmol/L), ALB (42.10 g/L vs. 39.70 g/L), UA (330.00 μmol/L vs. 299.05 μmol/L), Scr (65.10 μmol/L vs. 62.00 μmol/L), FI (43.70 pmol/L vs. 36.30 pmol/L), and Hb (138.00 g/L vs. 129.00 g/L), all statistically significant (all *P* ≤ 0.001). However, HDL-C levels were significantly lower in patients with NAFLD compared to those without (1.00 mmol/L vs. 1.10 mmol/L, *P* < 0.001). No statistically significant differences were observed between the two groups concerning gender, age, TC, LDL-C, ALP, TBIL, GFR, HbA1C, and PLT (*P* > 0.05). Notably, although the median waist circumference was identical between the two groups (87.00 cm), the quartiles of the NAFLD group were higher than those of the non-NAFLD group ([87.00 cm, 94.00 cm] vs. [80.00 cm, 87.00 cm]), with statistical significance between the two groups (*P* < 0.001).

**Table 1:** Baseline characteristics of T2DM patients with and without NAFLD.

Characteristics	T2DM without NAFLD (N = 1150)	T2DM with NAFLD (N = 1309)	P-value
Sex, n (%)			0.106
Male	641 (55.7)	773 (59.1)	
Female	509 (44.3)	536 (40.9)	
Age (years)	62.00 [54.00, 69.00]	61.00 [52.00, 70.00]	0.058
Height (cm)	162.00 [156.00, 166.00]	163.00 [157.00, 170.00]	<0.001
Weight (kg)	60.20 [52.40, 64.00]	67.00 [60.00, 75.50]	<0.001
BMI (kg/m²)	22.50 [20.57, 24.39]	25.34 [23.49, 27.48]	<0.001
Waist circumference (cm)	87.00 [80.00, 87.00]	87.00 [87.00, 94.00]	<0.001
SBP (mmHg)	131.00 [119.00, 142.00]	133.00 [123.00, 145.00]	<0.001
DBP (mmHg)	77.00 [72.00, 83.00]	78.00 [73.00, 86.00]	<0.001
Hypertension, n (%)			
YES	565 (49.1)	819 (62.6)	<0.001
Smoking, n (%)			
Never	865 (75.2)	1064 (81.3)	0.001
Former	86 (7.5)	79 (6.0)	
Current	199 (17.3)	166 (12.7)	
Alcohol consumption, n (%)			
YES	93 (8.1)	56 (4.3)	<0.001
Total cholesterol (mmol/L)	4.36 [3.59, 5.14]	4.39 [3.64, 5.20]	0.405
Triglycerides (mmol/L)	1.16 [0.84, 1.60]	1.66 [1.17, 2.53]	<0.001
HDL-C (mmol/L)	1.10 [0.91, 1.38]	1.00 [0.83, 1.19]	<0.001
LDL-C (mmol/L)	2.78 [2.09, 3.56]	2.75 [2.01, 3.45]	0.125
FBG (mmol/L)	6.96 [5.16, 9.95]	7.30 [5.74, 9.93]	<0.001
ALT (U/L)	16.00 [12.00, 23.00]	20.00 [14.00, 31.00]	<0.001
AST (U/L)	17.00 [14.00, 21.00]	18.00 [15.00, 24.00]	<0.001
ALP (U/L)	73.00 [58.25, 90.00]	73.00 [60.00, 88.00]	0.97
GGT (U/L)	18.00 [13.00, 29.00]	26.00 [17.00, 40.00]	<0.001
Total bilirubin (μmol/L)	8.60 [6.20, 11.80]	8.90 [6.50, 11.90]	0.21
Direct bilirubin (μmol/L)	3.00 [2.30, 4.10]	3.60 [2.70, 4.70]	<0.001
Total protein (g/L)	64.60 [61.10, 68.60]	66.70 [62.80, 70.30]	<0.001
Albumin (g/L)	39.70 [36.70, 42.10]	42.10 [39.50, 44.70]	<0.001
Globulin (g/L)	25.00 [22.80, 27.60]	24.40 [21.90, 26.90]	<0.001
Uric acid (μmol/L)	299.05 [242.00, 370.60]	330.00 [267.90, 400.00]	<0.001
Blood urea nitrogen (mmol/L)	5.78 [4.61, 7.31]	5.54 [4.49, 6.83]	0.002
Serum creatinine (μmol/L)	62.00 [48.92, 79.22]	65.10 [53.20, 81.00]	0.001
GFR (ml/min)	96.65 [82.00, 107.68]	95.51 [82.60, 106.38]	0.385
Fasting insulin (pmol/L)	36.30 [20.40, 44.55]	43.70 [38.40, 77.10]	<0.001
HbA1C (%)	8.50 [7.00, 11.00]	8.50 [7.00, 10.40]	0.051
White blood cells (10^9/L)	6.19 [5.13, 7.36]	6.51 [5.58, 7.87]	<0.001
Monocytes (10^9/L)	0.37 [0.30, 0.46]	0.39 [0.31, 0.49]	<0.001
Red blood cells (10^12/L)	4.27 [3.86, 4.65]	4.55 [4.24, 4.96]	<0.001
Hemoglobin (g/L)	129.00 [116.00, 140.00]	138.00 [127.00, 149.00]	<0.001
Platelets (10^9/L)	227.00 [188.00, 272.00]	231.00 [195.00, 275.00]	0.094
Diabetes complications, n (%)			
YES	754 (65.6)	261 (19.9)	<0.001
Cardio-cerebrovascular diseases, n (%)			
YES	141 (12.3)	92 (7.0)	<0.001
Respiratory system diseases, n (%)			
YES	65 (5.7)	8 (0.6)	<0.001

Values are presented as median [interquartile range] or number (percentage). Between-group comparisons were performed using the Wilcoxon rank-sum test for continuous variables and the χ² test for categorical variables. *P* < 0.05 was considered statistically significant.

BMI, body mass index; SBP, systolic blood pressure; DBP, diastolic blood pressure; HDL-C, high-density lipoprotein cholesterol; LDL-C, low-density lipoprotein cholesterol; FBG, fasting blood glucose; ALT, alanine aminotransferase; AST, aspartate aminotransferase; ALP, alkaline phosphatase; GGT, gamma-glutamyltransferase; GFR, glomerular filtration rate; HbA1c, glycated hemoglobin; RBC, red blood cell count; FI, fasting insulin; PLT, platelet count.

### 3.2 Feature selection

**Figure 2:**
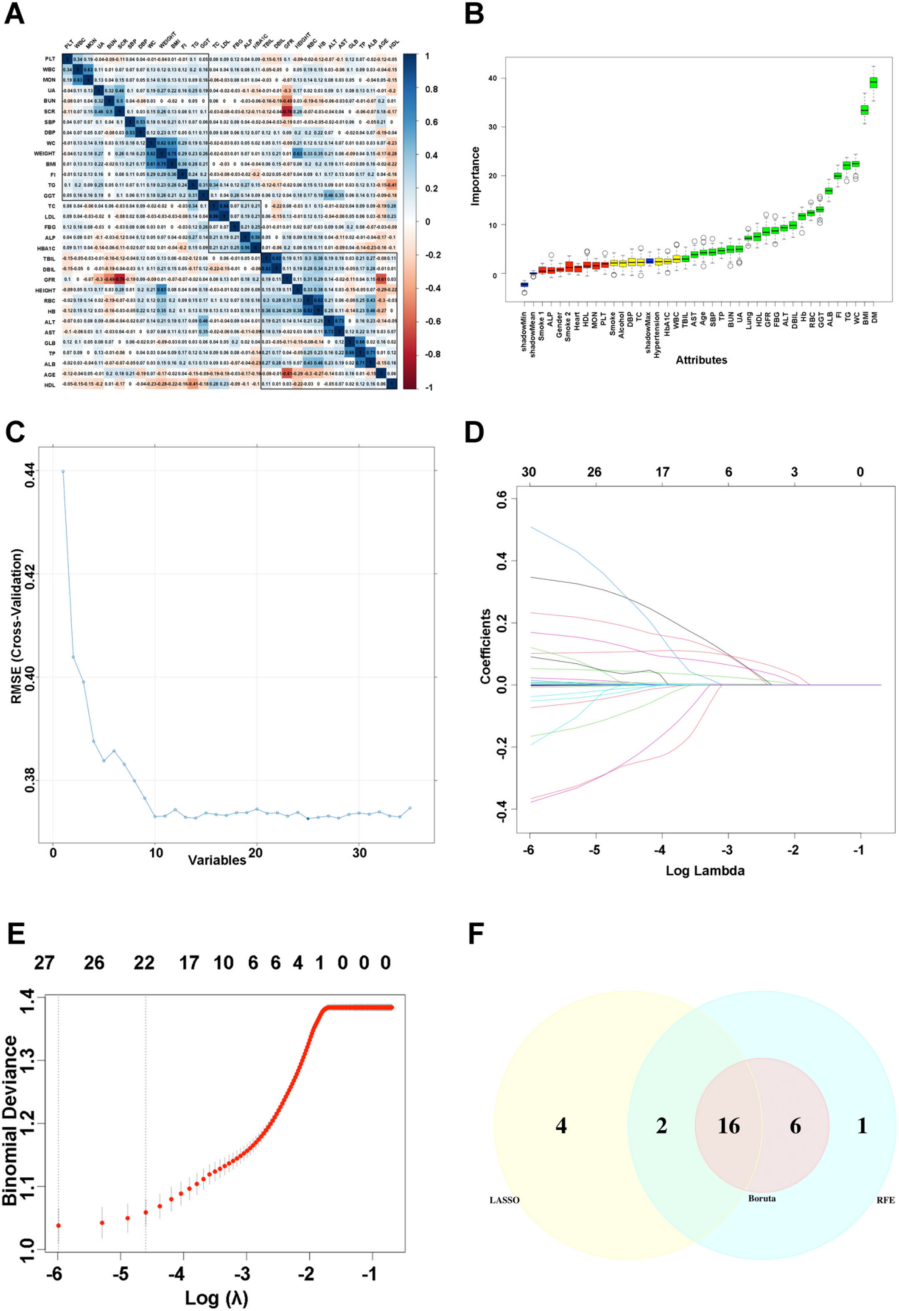
Feature selection using Boruta, RFE, and LASSO algorithms. **(A)** Correlation heatmap of all variables, with variables showing correlations >0.6 removed. **(B)** Boruta feature-selection results, where green indicates confirmed features, yellow tentative features, and gray unimportant features. **(C)** Recursive Feature Elimination (RFE) identifying the top-ranked predictors. **(D)** LASSO coefficient profiles for the 35 variables. **(E)** Selection of optimal lambda using 10-fold cross-validation. **(F)** Venn diagram summarizing the intersected predictors identified by all three methods.

Initially, variables with correlation coefficients exceeding 0.6 were excluded from the study (**Figure 2A**). Subsequently, three feature selection algorithms, Boruta (**Figure 2B**), RFE (**Figure 2C**), and LASSO regression (**Figures 2D, E**), were implemented on the training dataset to identify the most relevant variables. Ultimately, 16 predictive variables (**Figure 2F**) were chosen for subsequent model construction, including BMI, WC, SBP, TG, HDL-C, ALT, GGT, TBIL, DBIL, TP, ALB, BUN, GFR, FI, RBC, and Hb.

### 3.3 Model performance comparisons

Eight machine learning algorithms, including Logistic Regression (LG), Random Forest (RF), Decision Tree (DT), Gradient Boosting Machine (GBM), Extreme Gradient Boosting (XGBoost), k-Nearest Neighbors (k-NN), Support Vector Machine (SVM), and Naïve Bayes (NB), were utilized to predict NAFLD in Type 2 Diabetes Patients. Sensitivity, specificity, F1 score, AUC, positive predictive value (PPV), negative predictive value (NPV), positive likelihood ratio (LR+), and negative likelihood ratio (LR-) were evaluated using 10-fold cross-validation with five repeats in the training dataset, and the final tuned models were then assessed in the validation dataset.

**Figure 3:**
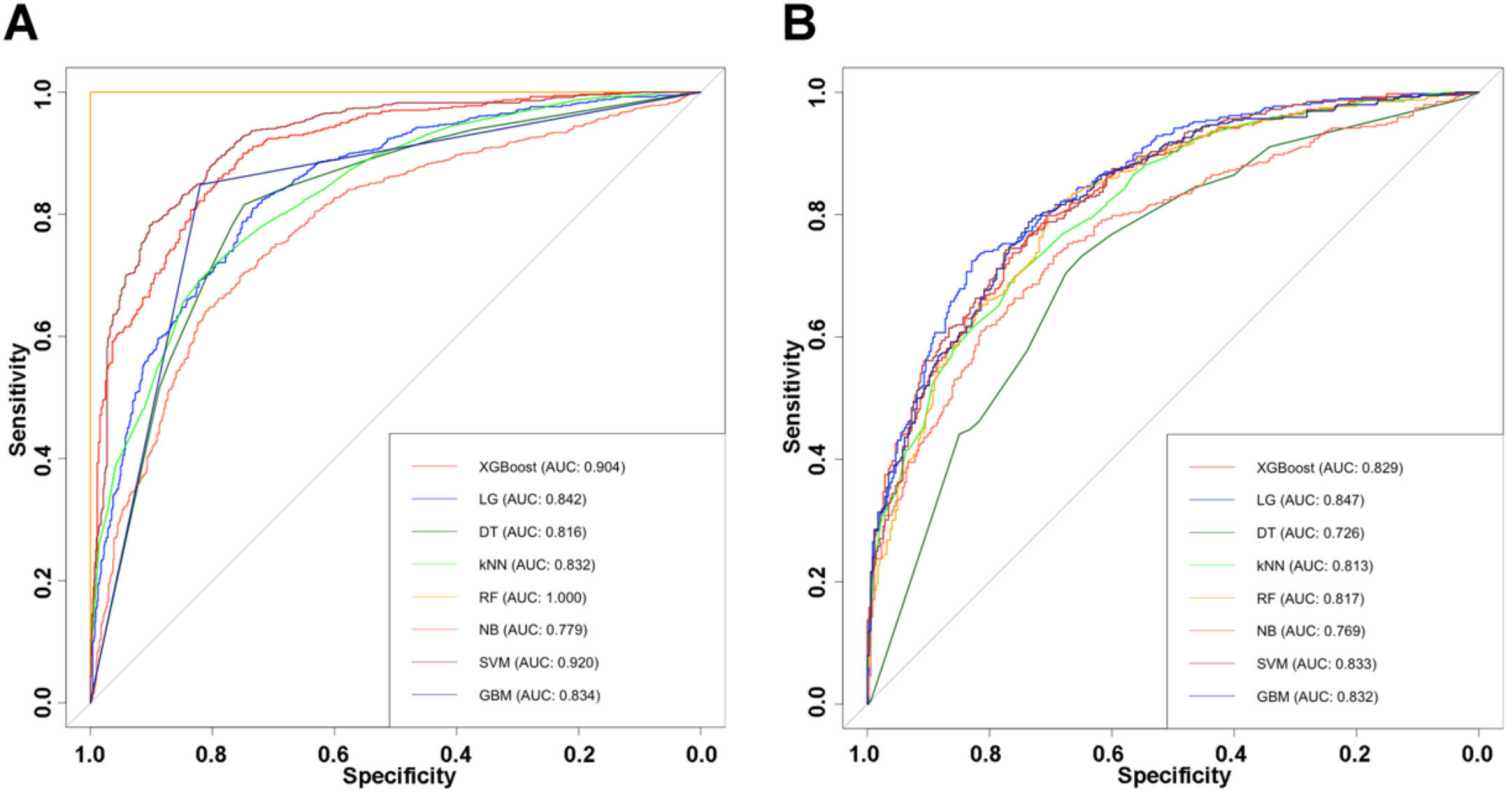
ROC curves of eight machine learning models for predicting NAFLD risk in type 2 diabetes patients in the training set **(A)** and validation set **(B)**.

**Figure 3** illustrates the discriminative performance of the eight models through ROC curves. Detailed performance metrics for these models are presented in **Tables 2**, **3**.

**Table 2:** Comparison of predictive performance of eight machine learning models in the training set.

Model	Sensitivity	Specificity	Accuracy	AUC (95% CI)	Recall	F1 Score	PPV	NPV	LR+	LR-	*P-value*
SVM	0.787	0.898	0.839	0.920(0.907-0.933)	0.787	0.839	0.898	0.791	7.700	0.237	–
LG	0.809	0.734	0.774	0.842(0.823-0.860)	0.809	0.792	0.776	0.771	3.041	0.260	<0.001
DT	0.816	0.748	0.784	0.816(0.796-0.836)	0.816	0.801	0.787	0.781	3.238	0.246	<0.001
k-NN	0.721	0.793	0.754	0.832(0.813-0.851)	0.721	0.758	0.798	0.712	3.483	0.352	<0.001
RF	1.000	1.000	1.000	1.000	1.000	1.000	1.000	1.000	–	0.000	<0.001
NB	0.705	0.749	0.725	0.779(0.757-0.801)	0.705	0.732	0.762	0.687	2.801	0.394	<0.001
XGBoost	0.900	0.750	0.832	0.904(0.886-0.923)	0.900	0.855	0.814	0.875	3.600	0.133	0.167
GBM	0.848	0.820	0.835	0.834(0.817-0.852)	0.848	0.846	0.843	0.825	4.711	0.185	<0.001

Values represent classification metrics for each model evaluated on the training set. AUC values are presented with 95% confidence intervals. Comparisons of AUCs between the SVM model and other models were performed using DeLong’s test. “-” indicates not applicable.

SVM, support vector machine; LG, logistic regression; DT, decision tree; k-NN, k-nearest neighbors; RF, random forest; NB, naïve Bayes; GBM, gradient boosting machine; AUC, area under the receiver operating characteristic curve; PPV, positive predictive value; NPV, negative predictive value; LR+, positive likelihood ratio; LR-, negative likelihood ratio.

**Table 3:** Predictive performance of eight machine learning models in the validation set.

Model	Sensitivity	Specificity	Accuracy	AUC (95% CI)	Recall	F1 Score	PPV	NPV	LR+	LR-	*P-value*
SVM	0.651	0.826	0.733	0.833(0.805-0.862)	0.651	0.721	0.810	0.672	3.740	0.423	–
LG	0.724	0.829	0.773	0.847(0.819-0.874)	0.724	0.773	0.828	0.725	4.210	0.333	0.127
DT	0.732	0.649	0.693	0.726(0.690-0.762)	0.732	0.718	0.703	0.680	2.080	0.413	<0.001
k-NN	0.694	0.762	0.726	0.813(0.782-0.843)	0.694	0.729	0.768	0.685	2.916	0.402	0.012
RF	0.806	0.704	0.759	0.817(0.787-0.848)	0.806	0.780	0.756	0.762	2.723	0.275	0.074
NB	0.732	0.696	0.715	0.769(0.735-0.803)	0.732	0.731	0.732	0.696	2.408	0.385	<0.001
XGBoost	0.799	0.707	0.756	0.829(0.800-0.858)	0.799	0.777	0.756	0.752	2.721	0.284	0.661
GBM	0.788	0.736	0.764	0.832(0.803-0.861)	0.788	0.780	0.773	0.753	2.977	0.288	0.860

Values represent classification metrics for each model evaluated in the validation set. AUC values are presented with 95% confidence intervals. Comparisons of AUCs between the SVM model and other models were performed using DeLong’s test.

Same abbreviations as **Table 2**.

In the training set, comparison of predictive performance across the eight machine learning models (**Table 2**) showed that the SVM model achieved the best overall results. It demonstrated the highest AUC (0.920, 95% CI: 0.907-0.933), along with strong accuracy (0.839), specificity (0.898), and F1 score (0.839); a PPV of 0.898, NPV of 0.791, LR+ of 7.700, and LR- of 0.237; indicating a favorable balance among discrimination, calibration, and sensitivity. DeLong’s test further confirmed that SVM significantly outperformed most of the other models (*P* < 0.001), with the exception of XGBoost (*P* = 0.167). Although the Random Forest model achieved perfect performance in the training set (all metrics = 1.000), this was considered indicative of overfitting rather than true predictive superiority. Given its overly optimistic training performance and lack of generalizable behavior, RF was not regarded as a suitable candidate for the optimal model. Overall, SVM exhibited the strongest and most reliable performance in the training dataset, establishing it as the leading model prior to validation testing.

**Figure 4:**
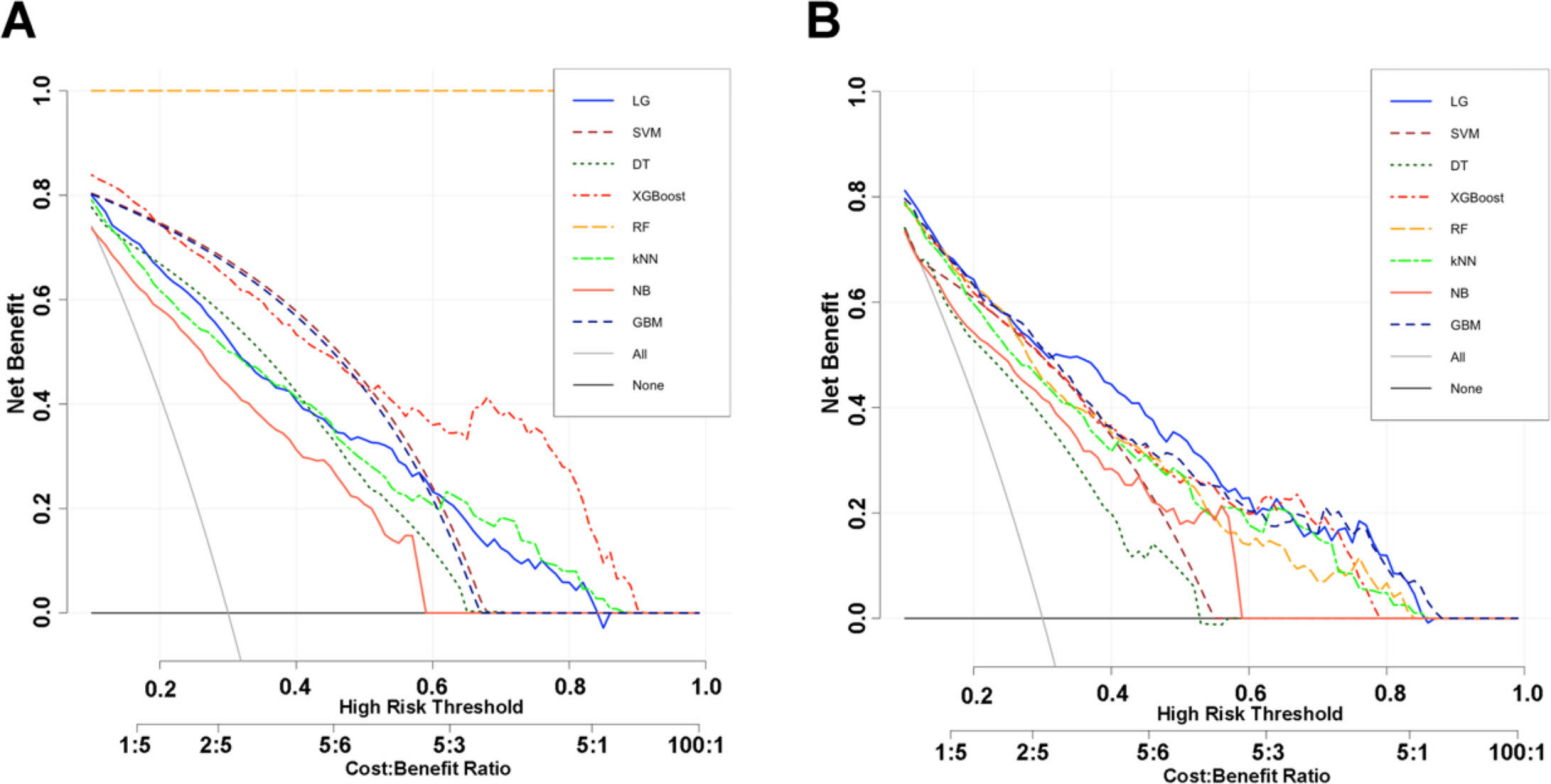
Decision curve analysis of eight machine learning models for predicting NAFLD risk in Type 2 Diabetes Patients in the training set **(A)** and validation set **(B)**.

In the validation set (**Table 3**), the SVM model demonstrated stable and robust predictive performance, with an AUC of 0.833 (95% CI: 0.805-0.862). Although its AUC was slightly lower than that of the logistic regression model, the difference was not statistically significant (*P* = 0.127). SVM also maintained a favorable balance among key classification metrics, with a specificity of 0.826, PPV of 0.810, NPV of 0.672, LR+ of 3.740, LR- of 0.423, sensitivity of 0.651, and F1 score of 0.721. Decision curve analysis further showed that SVM, along with the other models, provided a net clinical benefit across a broad range of threshold probabilities (**Figure 4**).

Integrating its superior performance in the training set with its consistent generalizability in the validation set, SVM achieved the most favorable overall balance among discrimination, calibration, and classification metrics. Therefore, SVM was selected as the optimal model for predicting NAFLD in patients with T2DM.

## 4 Discussion

Early identification of non-alcoholic fatty liver disease (NAFLD) in individuals with type 2 diabetes mellitus (T2DM) remains a substantial clinical challenge. Although liver biopsy and imaging modalities can diagnose hepatic steatosis, their invasiveness, operator dependence, and limited sensitivity for mild steatosis restrict their utility for large-scale screening. In contrast, demographic characteristics, anthropometric indices, and routinely measured biochemical parameters are readily available across diverse healthcare settings, making them practical candidates for constructing simple, objective NAFLD prediction tools. In this context, machine learning (ML) approaches offer unique advantages in leveraging multidimensional clinical data to enhance NAFLD risk stratification among high-risk populations such as individuals with T2DM.

Previous studies have illustrated the clinical relevance of routinely available biomarkers in NAFLD assessment. Yu et al (9). identified the diagnostic utility of serum hemoglobin for NAFLD. Chang et al. (10), in a prospective cohort of healthy Korean men whose ALT and GGT levels were within the reference intervals, demonstrated that modest elevations in ALT, even within normal limits, independently predicted the development of NAFLD. Miyake et al. (11) further reported that metabolic disease markers and ALT levels were independently associated with NAFLD and proposed revised ALT cutoff values to improve early identification. These findings collectively support the concept that simple biochemical measurements can provide meaningful insight into NAFLD risk.

In our study, ML-based models effectively predicted NAFLD in patients with T2DM. Compared with traditional regression-based approaches, which assume linear relationships and may overlook complex metabolic interactions, ML algorithms are capable of modeling nonlinear and high-dimensional patterns that more accurately reflect the multifactorial pathogenesis of NAFLD. Among the eight models evaluated, the support vector machine (SVM) yielded the most robust and generalizable performance in both the training and validation sets. This suggests that ML techniques hold promise for developing clinically practical tools that can optimize NAFLD screening efforts in T2DM populations. Consistent with previous findings by Miyake et al. (11), our feature selection procedures repeatedly identified BMI, waist circumference, and triglycerides (TG) as key predictors across models, reinforcing their central role in metabolic dysregulation underlying NAFLD.

Obesity is a well-established independent risk factor for both T2DM and NAFLD (2). Elevated BMI and visceral adiposity substantially increase NAFLD susceptibility by driving metabolic syndrome and related complications (12). Waist circumference, as a surrogate for visceral adiposity, further reflects cardiometabolic burden. In line with these mechanisms, our findings showed that T2DM patients with NAFLD had significantly higher BMI and waist circumference compared with those without NAFLD, underscoring the strong and consistent link between adiposity and NAFLD development in diabetic individuals.

Dyslipidemia represents another central metabolic pathway linking T2DM to NAFLD. NAFLD is characterized by hepatic and systemic lipid accumulation, and abnormal lipid metabolism plays a pivotal role in disease onset and progression (13). Our study identified elevated TG as a significant predictor of NAFLD in T2DM patients. This observation aligns with prior reports showing that NAFLD frequently presents with hypertriglyceridemia, reduced HDL-C, increased large VLDL particles, and higher concentrations of small, dense LDL-C (14, 15). These lipid alterations may partly result from increased cholesterol ester transfer protein activity (16). Large epidemiological studies have further demonstrated that higher lipid ratios, such as TC: HDL or TG: HDL, are strongly associated with more advanced NAFLD and greater risk of T2DM (17). HDL-C may also influence glucose metabolism by enhancing insulin secretion from pancreatic β-cells, highlighting its additional metabolic relevance (18).

In addition to lipid markers, bilirubin fractions have been explored as potential indicators of NAFLD. Some studies have reported inverse associations between direct bilirubin (DBIL) and NAFLD risk, suggesting that DBIL may exert protective metabolic effects independent of classical risk factors (19, 20). However, evidence regarding total bilirubin (TBIL) has been less consistent, possibly due to biochemical differences between conjugated and unconjugated fractions. Interestingly, in our T2DM cohort, DBIL showed a positive association with NAFLD, while TBIL demonstrated an inverse association, a pattern opposite to that observed in nondiabetic populations. This discrepancy may reflect altered bilirubin metabolism and greater metabolic heterogeneity among individuals with T2DM. Further research is warranted to elucidate these divergent associations.

Liver enzymes, particularly ALT and GGT, also emerged as important predictors in our models. Transaminase levels frequently fluctuate in NAFLD, and a substantial proportion of patients may show normal or only mildly elevated ALT or AST levels, typically up to 1.5–2 times the upper limit of normal (21). Elevated ALT has been linked to impaired hepatic insulin signaling and increased hepatic insulin resistance (22), mechanisms that closely relate to NAFLD pathophysiology. GGT, a membrane-bound enzyme involved in maintaining glutathione homeostasis and oxidation–reduction balance, has been strongly associated with hepatic steatosis and fibrosis (23, 24). Recent evidence indicates that higher GGT levels independently predict NAFLD and modify metabolic risk patterns (25, 26), further supporting their relevance in non-invasive assessment strategies.

Despite the strong predictive performance achieved by our models, several limitations warrant consideration. First, NAFLD diagnosis in this study relied on abdominal ultrasonography, which is less sensitive than liver biopsy and cannot reliably assess disease severity. Second, our analysis was based on a single-center cross-sectional cohort, and external validation in independent populations is necessary to confirm generalizability. Third, all participants were hospitalized patients, which may lead to selection bias and limit the generalizability of the model to outpatient or community based T2DM populations. Model performance may differ in non-hospitalized settings and requires further external validation in broader populations.

In summary, this study demonstrates that ML models based on routine clinical and biochemical indicators can effectively predict NAFLD risk in individuals with T2DM. The SVM model, in particular, exhibited strong discrimination and high clinical applicability. These findings support the potential value of ML-based approaches to enhance early NAFLD detection and risk stratification in diabetic populations, thereby contributing to improved metabolic management and long-term outcomes.

## Data Availability

The original contributions presented in the study are included in the article/supplementary material. Further inquiries can be directed to the corresponding authors.

## References

[1] Chalasani N Younossi Z Lavine JE Charlton M Cusi K Rinella M . The diagnosis and management of nonalcoholic fatty liver disease: Practice guidance from the American Association for the Study of Liver Diseases. Hepatology. (2018) 67:328–57. doi:10.1002/hep.29367. PMID: 28714183

[2] Younossi ZM Golabi P de Avila L Paik JM Srishord M Fukui N . The global epidemiology of NAFLD and NASH in patients with type 2 diabetes: A systematic review and meta-analysis. J Hepatol. (2019) 71:793–801. doi:10.1016/j.jhep.2019.06.021. PMID: 31279902

[3] Nair GG Tzanakakis ES Hebrok M . Emerging routes to the generation of functional β-cells for diabetes mellitus cell therapy. Nat Rev Endocrinol. (2020) 16:506–18. doi:10.1016/j.mayocp.2019.11.016. PMID: 32587391 PMC9188823

[4] Zheng Y Ley SH Hu FB . Global aetiology and epidemiology of type 2 diabetes mellitus and its complications. Nat Rev Endocrinol. (2018) 14:88–98. doi:10.1038/nrendo.2017.151. PMID: 29219149

[5] Kramer CK Retnakaran R . Liver enzymes and type 2 diabetes: a complex two-way relationship. J Diabetes Complicat. (2013) 27:301–2. doi:10.1016/j.jdiacomp.2013.04.009. PMID: 23684419

[6] Fruci B Giuliano S Mazza A Malaguarnera R Belfiore A . Nonalcoholic fatty liver: a possible new target for type 2 diabetes prevention and treatment. Int J Mol Sci. (2013) 14:22933–66. doi:10.3390/ijms141122933. PMID: 24264040 PMC3856099

[7] Cao YT Xiang LL Qi F Zhang YJ Chen Y Zhou XQ . Accuracy of controlled attenuation parameter (CAP) and liver stiffness measurement (LSM) for assessing steatosis and fibrosis in non-alcoholic fatty liver disease: A systematic review and meta-analysis. EClinicalMedicine. (2022) 51:101547. doi:10.1016/j.eclinm.2022.101547. PMID: 35844772 PMC9284399

[8] National Workshop on Fatty Liver and Alcoholic Liver Disease, Chinese Society of Hepatology, Chinese Medical Association; Fatty Liver Expert Committee, Chinese Medical Doctor Association . Guidelines of prevention and treatment for nonalcoholic fatty liver disease: a 2018 update. Zhonghua Gan Zang Bing Za Zhi. (2018) 26:195–203. doi: 10.3760/cma.j.issn.1007-3418.2018.03.008. PMID: 29804393 PMC12769340

[9] Yu C Xu C Xu L Yu J Miao M Li Y . Serum proteomic analysis revealed diagnostic value of hemoglobin for nonalcoholic fatty liver disease. J Hepatol. (2012) 56:241–7. doi:10.1016/j.jhep.2011.05.027. PMID: 21756851

[10] Chang Y Ryu S Sung E Jang Y . Higher concentrations of alanine aminotransferase within the reference interval predict nonalcoholic fatty liver disease. Clin Chem. (2007) 53:686–92. doi:10.1373/clinchem.2006.081257. PMID: 17272484

[11] Miyake T Kumagi T Hirooka M Koizumi M Furukawa S Ueda T . Metabolic markers and ALT cutoff level for diagnosing nonalcoholic fatty liver disease: a community-based cross-sectional study. J Gastroenterol. (2012) 47:696–703. doi:10.1007/s00535-012-0534-y. PMID: 22331365

[12] Byrne CD Targher G . NAFLD: a multisystem disease. J Hepatol. (2015) 62:S47–64. doi:10.1016/j.jhep.2014.12.012. PMID: 25920090

[13] Jarvis H Craig D Barker R Spiers G Stow D Anstee QM . Metabolic risk factors and incident advanced liver disease in non-alcoholic fatty liver disease (NAFLD): A systematic review and meta-analysis of population-based observational studies. PloS Med. (2020) 17:e1003100. doi:10.1371/journal.pmed.1003100. PMID: 32353039 PMC7192386

[14] Männistö VT Simonen M Soininen P Tiainen M Kangas AJ Kaminska D . Lipoprotein subclass metabolism in nonalcoholic steatohepatitis. J Lipid Res. (2014) 55:2676–84. doi:10.1194/jlr.P054387, PMID: 25344588 PMC4242459

[15] DeFilippis AP Blaha MJ Martin SS Reed RM Jones SR Nasir K . Nonalcoholic fatty liver disease and serum lipoproteins: the Multi-Ethnic Study of Atherosclerosis. Atherosclerosis. (2013) 227:429–36. doi:10.1016/j.atherosclerosis.2013.01.022. PMID: 23419204 PMC4049078

[16] Heeren J Scheja L . Metabolic-associated fatty liver disease and lipoprotein metabolism. Mol Metab. (2021) 50:101238. doi:10.1016/j.molmet.2021.101238. PMID: 33892169 PMC8324684

[17] Wu KT Kuo PL Su SB Chen YY Yeh ML Huang CI . Nonalcoholic fatty liver disease severity is associated with the ratios of total cholesterol and triglycerides to high-density lipoprotein cholesterol. J Clin Lipidol. (2016) 10:420–5.e1. doi:10.1016/j.jacl.2015.12.026. PMID: 27055973

[18] Femlak M Gluba-Brzózka A Ciałkowska-Rysz A Rysz J . The role and function of HDL in patients with diabetes mellitus and the related cardiovascular risk. Lipids Health Dis. (2017) 16:207. doi:10.1186/s12944-017-0594-3. PMID: 29084567 PMC5663054

[19] Kunutsor SK Frysz M Verweij N Kieneker LM Bakker SJL Dullaart RPF . Circulating total bilirubin and risk of non-alcoholic fatty liver disease in the PREVEND study: observational findings and a Mendelian randomization study. Eur J Epidemiol. (2020) 35:123–37. doi:10.1007/s10654-019-00589-0. PMID: 31773475 PMC7125247

[20] Tian J Zhong R Liu C Tang Y Gong J Chang J . Association between bilirubin and risk of Non-Alcoholic Fatty Liver Disease based on a prospective cohort study. Sci Rep. (2016) 6:31006. doi:10.1038/srep31006. PMID: 27484402 PMC4975069

[21] Gao X Fan JG . Diagnosis and management of non-alcoholic fatty liver disease and related metabolic disorders: consensus statement from the Study Group of Liver and Metabolism, Chinese Society of Endocrinology. J Diabetes. (2013) 5:406–15. doi: 10.1111/1753-0407.12056, PMID: 23560695 PMC3933762

[22] Gómez-Sámano MA Cuevas-Ramos D Mehta R Brau-Figueroa H Meza-Arana CE Gulias-Herrero A . Association of Alanine Aminotransferase Levels (ALT) with the Hepatic Insulin Resistance Index (HIRI): a cross-sectional study. BMC Endocr Disord. (2012) 12:16. doi: 10.1186/1472-6823-12-16, PMID: 22947097 PMC3515498

[23] Chen LW Huang MS Shyu YC Chien RN . Gamma-glutamyl transpeptidase elevation is associated with metabolic syndrome, hepatic steatosis, and fibrosis in patients with nonalcoholic fatty liver disease: A community-based cross-sectional study. Kaohsiung J Med Sci. (2021) 37:819–27. doi:10.1002/kjm2.12395. PMID: 34002481 PMC11896555

[24] Xing Y Chen J Liu J Ma H . Associations between GGT/HDL and MAFLD: A cross-sectional study. Diabetes Metab Syndr Obes. (2022) 15:383–94. doi:10.2147/dmso.s342505. PMID: 35177915 PMC8843704

[25] Hossain IA Rahman Shah MM Rahman MK Ali L . Gamma glutamyl transferase is an independent determinant for the association of insulin resistance with nonalcoholic fatty liver disease in Bangladeshi adults: Association of GGT and HOMA-IR with NAFLD. Diabetes Metab Syndr. (2016) 10:S25–9. doi:10.1016/j.dsx.2015.09.005. PMID: 26482965

[26] Oni ET Figueredo V Aneni E Veladar E McEvoy JW Blaha MJ . Non-alcoholic fatty liver disease modifies serum gamma-glutamyl transferase in cigarette smokers. J Clin Med Res. (2020) 12:472–82. doi:10.14740/jocmr3932. PMID: 32849935 PMC7430878

